# Hypertriglyceridemia Therapy: Past, Present and Future Perspectives

**DOI:** 10.3390/ijms25179727

**Published:** 2024-09-08

**Authors:** Ileana Canfora, Sabata Pierno

**Affiliations:** Section of Pharmacology, Department of Pharmacy and Drug Sciences, University of Bari “Aldo Moro”, 70121 Bari, Italy; ileana.canfora@uniba.it

**Keywords:** hypertriglyceridemia, cardiovascular disease, fibrates, lipid-lowering drugs, pharmacotherapy

## Abstract

Hypertriglyceridemia therapy is essential for preventing cardiovascular diseases. Fibrates belong to an important class of lipid-lowering drugs useful for the management of dyslipidaemia. By acting on the peroxisome proliferator-activated receptor (PPAR)-α, these drugs lower serum triglyceride levels and raise high-density lipoprotein cholesterol. Fibrate monotherapy is associated with a risk of myopathy and this risk is enhanced when these agents are administered together with statins. However, whereas gemfibrozil can increase plasma concentrations of statins, fenofibrate has less influence on the pharmacokinetics of statins. Pemafibrate is a new PPAR-α-selective drug considered for therapy, and clinical trials are ongoing. Apart from this class of drugs, new therapies have emerged with different mechanisms of action to reduce triglycerides and the risk of cardiovascular diseases.

## 1. Introduction

Cardiovascular diseases (CVDs) are pathological conditions responsible for morbidity and mortality worldwide. Elevated levels of triglycerides (TGs) and low density lipoprotein cholesterol (LDL), together with low levels of high density lipoprotein cholesterol (HDL), represent risk factors. The main treatment for CVD risk reduction is the lowering of LDL cholesterol (LDL-C), mostly in very high-risk patients [1]. For this reason, statins are considered the first-line therapeutical option for patients with an alteration in blood lipid levels [2]. In addition, drugs that may increase HDL cholesterol (HDL-C) are of importance in reducing the risk. The reduction of triglycerides is also considered a therapeutic need [3]. In patients with very high triglyceride levels, lowering them is required in light of the possible increased risk of pancreatitis. Fibrates are the most commonly used drugs for reducing triglyceride levels in the blood and avoiding the consequences of this pathological condition [4]. Fibrates interact with the peroxisome proliferator-activated receptors (PPARs), which belong to the nuclear hormone receptor superfamily. PPARs, by binding to PPAR response regulatory elements (PPRE), heterodimerize with the retinoid X receptor (RXR) and modulate the expression of genes involved in adipogenesis, lipid metabolism, inflammation and carbohydrate metabolism. In this review, we analyse the pharmacological aspects of these drugs as well as new therapeutic proposals by studying the past, present and future of the treatment of hypertriglyceridemia. It should also be noted that a concomitant hypolipidemic diet is necessary to support therapy.

## 2. Past Therapies: Fibrates and Niacin

### 2.1. Fibrates: Mechanism of Action, Side Effects and Interaction with Other Drugs

Fibrates were discovered before statins and used to treat hypercholesterolemia, with poor benefits. The first molecule in therapy was clofibrate, developed in the 1960s [5,6]. Clofibrate is now no longer available because of the risk of adverse effects. By acting on the peroxisome proliferator-activated receptor (PPAR)-α, the fibrates lower serum triglyceride levels by 20–40% and increase high-density lipoproteins by 5–20% [7]. They are used to reduce the risk of heart disease, heart attack and stroke in people with high triglyceride levels in their blood.

These drugs can be used as monotherapy or associated with statins in the treatment of mixed hyperlipidaemias; however, this therapy needs particular attention because of the possible side effects. Statins and fibrate are generally well tolerated; however, they can potentially produce serious adverse effects in a small portion of the population, especially on skeletal muscle tissue. Statin-associated muscle disorders range from myalgia to severe myopathy with muscle weakness and an increase in serum creatine kinase (CK), resulting in rhabdomyolysis; however, life-threatening rhabdomyolysis with muscle necrosis and electrolyte alteration, myoglobinuria and renal failure is a rare event [8]. The risk of rhabdomyolysis with cerivastatin monotherapy was greater compared to other statins, and even higher when cerivastatin was administered in combination with gemfibrozil [9]. Indeed, because of massive muscle fibre necrosis, the occurrence of fatal acute renal failure was possible. For this reason, cerivastatin was withdrawn from the market in 2001 [10].

Different studies have shown that statins and fibrate can modify gene and protein expression and activity in skeletal muscle, causing severe abnormalities of its function and clear damage [11,12,13,14]. For instance, an upregulation of the ryanodine receptor, suggestive of intracellular calcium increase, was found in muscle biopsies of statin-treated patients showing evident structural damage [15]. Our previous studies have demonstrated that statin can affect skeletal muscle function by modifying calcium homeostasis and resting chloride conductance (gCl). Lipophilic statins increased resting cytosolic calcium either after in vivo treatment in rats or following in vitro application on skeletal muscle fibres. Further studies revealed that the calcium increase was not due to an increase in sarcolemma permeability to calcium but to its release from the mitochondria [11,16,17]. This release leads to a consequent large delivery of calcium from the sarcoplasmic reticulum, the cellular compartment in which calcium is stored. The alteration in calcium homeostasis is thus responsible for the alteration in contractile function. These drugs also reduced resting gCl [11,18,19,20], a parameter sustained by the ClC-1 chloride channel that is negatively modulated by protein kinase C (PKC) [21,22,23]. This parameter is normally high in fast-twitch muscles because PKC is almost inactive under resting conditions. This is important to guarantee physiological muscle cell membrane potential and excitability [24,25,26,27,28]. The reduction of gCl by fibrates is responsible for the increase in sarcolemma excitability. In addition, fenofibrate reduced ClC-1 expression in the skeletal muscle of chronically treated rats, likely through PPAR activation. To explore the mechanism through which the fibrates affect gCl, we performed patch-clamp experiments evaluating the activity of the human ClC-1 channel expressed in cultured HEK cells before and after the application of fenofibric acid. We found that this drug was able to directly inhibit ClC-1 chloride channel activity [11]. Fenofibric acid caused a rapid and marked decrease in inward hClC-1 currents and acceleration of current deactivation. Fenofibric acid is capable of interfering with ClC-1 gating by interacting with an intracellular binding site [11]. In support of this finding, the in vitro application of chelerythrine, a PKC inhibitor, to EDL muscles of rats treated with fenofibrate did not antagonize the reduction in gCl, as occurs in statin-treated animals [11].

It should be noted that the reduction in gCl induced by statin and fenofibrate in rats at doses higher than those used in clinical settings was not greater than 30%, demonstrating milder symptoms. Indeed, myotonia-like phenomena are characterized by a stronger reduction in gCl (more than 50% of the normal value) [27]. Accordingly, the electromyographic activity recorded on fluvastatin-, atorvastatin- and fenofibrate-treated animals showed signs of altered excitability characterized by spontaneous brief bursts of action potentials (AP) [11], similar to those recorded in humans during muscle cramping or fasciculation [29,30]. In the fenofibrate-treated rat, repetitive high-frequency discharges persisted for 4 s. It is possible that the reduction in gCl observed following drug exposure may be responsible for the mild adverse effects of hypolipidemic drugs, such as myalgia and cramps [11,18].

Moreover, our previous studies demonstrated that the modification of the fibrate molecule in a series of newly synthesized 2-aryloxy-3-phenyl-propanoic acids with improved potency on PPARα showed a more beneficial pharmacological profile compared to fibrates currently used in therapy [31,32]. This supports the possibility of improving the pharmacological characteristics of these drugs and sustaining the research for new compounds.

**Figure 1:**
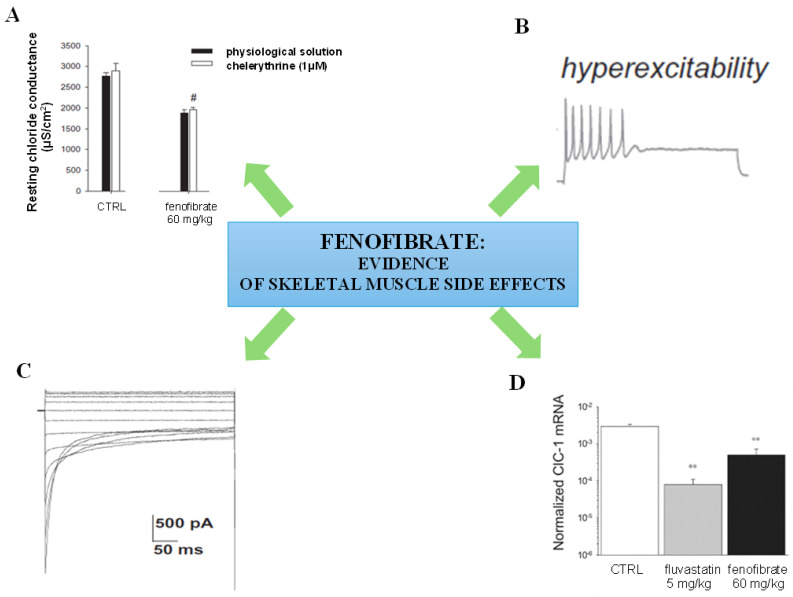
Evidence of fenofibrate-induced side effects on skeletal muscle function collected by previous studies [11]. (**A**) Fenofibrate administered in vivo to rats reduced the resting chloride conductance (gCl) sustained by the ClC-1 chloride channel in the extensor digitorum longus fast-twitch muscle. This effect is responsible for sarcolemma instability. Since ClC-1 is negatively modulated by protein kinase C (PKC), we evaluated the effects of the in vitro application of chelerythrine, an inhibitor of this protein. Chelerythrine did not restore gCl, suggesting that the reduction in gCl is not mediated by PKC activation but is a direct inhibitory effect on the channel. # Significantly different with respect to control (CTRL) value measured in physiological solution (by Student’s *t*-test) (*p* < 0.001) [11]. (**B**) Fenofibrate increased sarcolemma excitability, evidenced by the increase in the maximum number of spikes in rat skeletal muscle fibres [11]. (**C**) Effect of in vitro application of fenofibric acid on hClC-1 channel expression in cultured HEK293 cells. Whole-cell patch-clamp chloride currents were recorded in the absence and presence of 500 μM of fenofibric acid. Fenofibric acid significantly reduced inward chloride currents, sustaining the direct inhibitory effect on the channel [11]. (**D**) Effects of fluvastatin and fenofibrate chronic treatment on muscle ClC-1 mRNA expression. Normalized ClC-1 mRNA levels in the extensor digitorum longus muscle of fluvastatin- and fenofibrate-treated rats with respect to control (CTRL) rats were measured by real-time polymerase chain reaction. The levels of ClC-1 were normalized to β-actin, which was constant in all muscle preparations. Both fluvastatin and fenofibrate significantly reduced ClC-1 expression. ** Significantly different with respect to CTRL (*p* < 0.05, one-way ANOVA test followed by Tukey’s post-test) [11]. Adapted from Ref. [11].

As an example, Figure 1 summarizes the results obtained in our previous studies and hypothesizes the sequence of events involved in fenofibrate-induced ClC-1 channel impairment and skeletal muscle function alteration [11].

### 2.2. Niacin

Niacin should also be mentioned among the first drugs approved for the treatment of dyslipidaemia [33]. Niacin has been shown to decrease all pro-atherogenic lipid and lipoprotein particles. In 30 clinical studies with 4749 subjects, treatment with three niacin preparations (immediate-release, sustained-release or extended-release), was effective in reducing total cholesterol by 10%, TG by 20% and LDL-C by 14%, and increasing HDL-C by 16% [34]. Surprisingly, the reductions in LDL-C and TGs were greater in women than in men. This effect might be due to a smaller body mass in women, leading to increased circulating niacin levels. A number of studies have considered the addition of niacin to statin therapy. As expected, combined therapy results in further reductions in atherogenic lipoprotein particles and an increase in HDL-C levels. Despite these positive effects, this preparation is no longer widely used in many markets to treat dyslipidaemia because of an increased risk of serious liver toxicity due to toxic metabolite development and other side effects (Table 1). Additionally, this drug failed to show a significant decrease in atherosclerotic disease progression in clinical studies. It should be mentioned that acipimox, a niacin analogue, can be used in particular situations as an additional or alternative treatment for hyperlipoproteinemia to reduce high triglyceride levels (EMA/779546/2013) [35].

## 3. The Present: Currently Used Drugs, Nutraceuticals and Phytotherapy

The abnormal increase in triglyceride-rich lipoproteins, such as chylomicron remnants, very light density lipoprotein (VLDL) and intermediate density lipoprotein (IDL) can contribute to atherogenesis. The influence of these lipids in the genesis of atherosclerosis and cardiovascular diseases is likely indirect because they are not found in atherosclerotic plaque, but is likely due to the metabolism of cholesterol-carrying lipoproteins and the activation of inflammatory mechanisms. Thus, the pharmacological interventions that lower triglycerides may be useful in therapy. It has been observed that a slowly metabolized LDL is derived from the lipolysis of large VLDL species and has prolonged permanency in the blood [36]. Indeed, these LDLs strongly contribute to foam cell formation. It has been found that remnant particles can induce inflammation more potently than LDL, an effect that can be responsible for atherosclerosis [37]. In addition, it has been found that triglyceride concentrations are inversely correlated with HDL levels.

### 3.1. Fibrates

Fibrates are the most employed therapeutic option for controlling triglyceride levels in the blood. These drugs include gemfibrozil, bezafibrate, ciprofibrate and fenofibrate. Gemfibrozil, fenofibrate, bezafibrate and ciprofibrate are marketed in Europe, whereas only gemfibrozil and fenofibrate are available in the United States. Fenofibrate is the most used in clinical practice (Table 2). All fibrates work via activation of the nuclear hormone receptor PPARα. They are prodrugs that are transformed into active form in the liver. Among fibrates, fenofibrate is mainly active on PPARα, whereas bezafibrate acts on PPARα-β/δ and glitazones (a family of drugs used in the treatment of diabetes mellitus type 2) act on PPARγ. They can increase lipoprotein lipase expression and activity and break down triglycerides. Triglyceride concentrations depend in large part on the activity of the enzyme lipoprotein lipase, which is found on the surface of vascular endothelial cells. This enzyme releases free fatty acids from the triglycerides, reducing their concentration. Genetic variants that lower lipoprotein lipase activity increase triglyceride-rich lipoprotein concentrations and raise the risk of cardiovascular diseases. These drugs are also involved in the modulation of inflammatory factors, including tumor necrosis factor-α (TNF-α), nuclear factor kappa B (NF-κB) and cyclooxygenase-2 (COX-2) [38]. Fibrates are generally well tolerated, with mild to severe adverse effects such as gastrointestinal and liver disorders and cholelithiasis in a few patients. Fibrates are also associated with an increased risk of pancreatitis. However, a serious safety risk associated with fibrates is myopathy, and the risk can increase when these drugs are used in combination with statins. For instance, gemfibrozil inhibits statin metabolism by acting via the glucuronidation pathway, which leads to marked increases in plasma concentrations of statins. Fenofibrate does not share the same pharmacokinetic pathways, so the risk of myopathy is much less when it is combined with statins. Simvastatin is a substrate of cytochrome P450 3A4, and a consequence of its coadministration with potent inhibitors of cytochrome P450 3A4 is the increase in the concentration of the drug in the plasma and risk of myopathy and rhabdomyolysis [39]. It is authorized in the EU as a commercial preparation that contains fenofibrate and simvastatin. It should be noted that the most recent fibrates are also capable of partially reducing cholesterol levels in the blood, thus avoiding the combined use of fibrates and statins. Fibrates also increase HDL lipoprotein levels due to stimulation of apo A-I and apo A-II expression. Moreover, by acting on PPAR, fibrates downregulate apolipoprotein C-III (apo C-III), a protein that by inhibiting lipoprotein lipase elevates triglyceride-rich lipoproteins; thus, these compounds prevent the inhibition of the expression of the lipoprotein lipase gene and affects triglyceride metabolism.

It should also be mentioned that statins can reduce triglycerides other than LDL-C. Although their action is based on the reduction of blood cholesterol levels by reducing endogenous (liver-produced) cholesterol synthesis through competitive inhibition of the enzyme 3-hydroxy-3-methyl-Glutaryl-Coenzyme A (HMG-CoA) reductase, they are also effective in the treatment of mild hypertriglyceridemia. The active substances belonging to this group are atorvastatin, fluvastatin, lovastatin, pravastatin, rosuvastatin and pitavastatin, the latter of which is used only in limited countries.

### 3.2. Antisense Oligonucleotide

To find other effective and safe drugs able to reduce triglyceride-rich lipoprotein concentrations, other molecules showing different mechanisms of action have recently been designed. These drugs include volanesorsen, which is authorized in the European Union for the treatment of familial chylomicronaemia syndrome (FCS), a rare disease caused by impaired functioning of lipoprotein lipase. It is an antisense oligonucleotide administered subcutaneously that specifically blocks the transcription of apo C-III, which is mainly present in chylomicrons, VLDL, their remnants and HDL [40]. Apo C-III may also exert independent proinflammatory effects. In patients with hyperchylomicronaemia, volanesorsen lowered triglycerides by over 70%; however, it can cause injection-site reactions in almost 25% of patients. A new formulation of an antisense oligonucleotide conjugated with N-acetyl-galactosamine (GalNAc) that specifically targets apo C-III in the hepatocytes increases binding capacity and affinity and limits unwanted effects such as injection-site reactions and thrombocytopaenia. Clinical trials show the efficacy of volanesorsen in the reduction of blood fat levels and pancreatitis. The antisense oligonucleotide therapy strategy was found to be helpful; however, 14 patients taking volanesorsen did not finish the trial because of platelet reduction. In spite of this, EMA authorized volanesorsen for the treatment of FCS in consideration of the benefit induced by this therapy [34,41,42,43]. Based on the results of the APPROACH/COMPASS trials, volanesorsen was approved as a treatment for patients with genetically confirmed FCS in the European Union and United Kingdom (UK) in 2019, but approval was denied in the United States based on its risk–benefit ratio [44].

### 3.3. Human Monoclonal Antibody

A member of a new class of drugs is evinacumab, a human monoclonal antibody that neutralizes angiopoietin-like 3 (ANGPTL3). ANGPTL3 is an inhibitor of lipoprotein lipase and is responsible for the increase in plasma triglycerides and LDL cholesterol. It is authorized for use in the EU and USA. Treatment with evinacumab led to reduced plasma TGs and, surprisingly, LDL-C levels in patients [42,43,45]. Of particular interest is the observation that the inhibition of ANGPTL3 significantly reduced LDL-C in patients with homozygous familial hypercholesterolemia (HoFH), a genetic disease characterized by the mutation of both alleles of hepatic LDL receptor gene responsible for LDL internalization and degradation [46]. This mutation impairs the removal of LDL-C from the bloodstream, resulting in an uncontrolled increase in total cholesterol and LDL-C. For this reason, the drug is indicated in this severe pathology that until now lacked an efficient therapy. Indeed, statins, which largely depend on the activity of the LDL receptor, are ineffective in this condition.

### 3.4. Gene Therapy

Alipogene tiparvovec is a gene therapy approved in Europe for adult patients with Familial Lipoprotein Lipase Deficiency and a history of multiple or severe episodes of pancreatitis that have discontinued diet [34,47,48]. Alipogene tiparvovec is an adeno-associated virus gene therapy that results in the expression of the naturally occurring human lipoprotein lipase gene. In patients treated with alipogene tiparvovec, lipoprotein lipase expression was demonstrated to be present in muscle biopsies. It needs to be administered by multiple intramuscular injections. However, despite the significant triglyceride-lowering effect, alipogene tiparvovec is no longer clinically available because of the lack of long-term efficacy due to the development of anti-AVV-1 antibodies and its potential tumorigenic effect.

### 3.5. Fatty Acids

Omega-3s are compounds belonging to the group of fatty acids. Omega-3 fatty acids are called essential fatty acids because the body is not able to produce them. For this reason, they must be consumed in the diet. The precursor of omega-3 is alpha-linolenic acid (ALA), which is converted into eicosapentaenoic acid (EPA) and docosahexaenoic acid (DHA), the biologically active omega-3s [49]. In the GISSI study, 1 g/day of supplements was administered with a combination of DHA/EPA ethyl esters in a ratio of 2:1 [50,51]. This study demonstrated a decrease in TG levels and a protective effect in patients with myocardial infarction. There are four prescription products approved by the FDA that contain various amounts of EPA and DHA [52]. The non-blinded JELIS study in Japanese individuals, using a higher dose than most other studies (1.5 g/day of EPA), shows a significant reduction in the primary endpoint [53]. However, further studies are required because recent trials have produced disparate results; indeed, the reduction in lipids seems to be insufficient to prevent or treat CV events [54]. The differences observed in these studies could be due to variations in omega-3 fatty acid preparation, the ratio between the mixed components, the doses employed or the placebo. Preclinical studies in animal models have demonstrated that these compounds inhibit fatty acid synthesis and stimulate fatty acid oxidation in the liver, likely through PPARα activation. These effects are responsible for the reduction in fatty acid availability for TG synthesis. In addition, omega-3 fatty acid administration reduces apo C-III levels, contributing to an increase in the clearance of TG-rich lipoproteins [55,56]. In addition, they are responsible for anti-inflammatory and antioxidant properties, as well as beneficial effects on endothelial function [57]. Mild side effects such as diarrhoea, nausea, dyspepsia and abdominal discomfort have been induced with omega-3 therapy.

### 3.6. Nutraceuticals and Phytotherapeutics

Nutraceuticals and phytotherapeutics are also considered for hypertriglyceridemia therapy. Clinical studies have found that artichoke leaf extract (*Cynara scolymus*, *Cynara cardunculus*) has good hypolipidaemic and hepatoprotective effects due to mono- and dicaffeoylquinic acid (cynarin and chlorogenic acid), caffeic acid, volatile sesquiterpenes and flavonoids. The mechanisms underlying the hypolipidemic activity of artichoke appear to be dual: the interaction of luteolin with HMG-CoA reductase and the regulatory pathways of sterol regulatory element-binding proteins (SREBPs) and Acyl-Coenzyme A Cholesterol Acyltransferase (ACAT) in the liver [58]. A study of 702 subjects showed a significant reduction in plasma TG concentrations. In addition, artichoke extract showed an improvement in hepatic transaminases, fasting blood glucose and systolic blood pressure. Few side effects, mainly gastrointestinal, have been reported [59].

Berberine (BBR) is a quaternary benzylisoquinoline alkaloid found in the root, rhizome, stem, fruit and bark of several plant species such as Coptis (*Coptis chinensis*, *Coptis japonica*), Hydrastis (*Hydrastis canadensis*) and Berberis (*Berberis aristata*, *Berberis vulgaris*, *Berberis croatica*) [60]. BBR is an activator of AMP-activated protein kinase (AMPK), which activates fatty acid oxidation and inhibits the expression of lipogenic genes [61]. Berberine also improves lipid dysregulation in obesity. It is an inhibitor of NADPH-mediated oxidative stress. A recent clinical study of 130 patients undergoing percutaneous coronary intervention (PCI) randomised patients into two groups and treated them with BBR (600 mg/day) or placebo in addition to standard therapies. The BBR group showed a reduction in TG (26% BBR vs. 13% control: statistical significance was not reached due to large inter-individual variations) and LDL-C (24% vs. 17% BBR control: *p* < 0.001) compared to the control group [62].

Bergamot is the common name for the *Citrus bergamia* fruit. Its composition is particularly rich in flavonoids (such as neoeriocitrin, neohesperidine, naringin, rutin, neodesmin and roifolin) [63]. The effect of bergamot on dyslipidaemia has been observed. Some studies have demonstrated the hypolipidemic properties of bergamot: the polyphenols able to reduce LDL-C triglycerides, non-HDL-C, malonyl-dialdehyde, fasting plasma insulin, leptin, leptin/adiponectin ratio, C-reactive protein (CRP) and TNF-α, varying according to the degree of purification of the extract [64,65].

**Table 2:** The present: currently used drugs for hypertriglyceridemia.

Drug	Year of Approval	Mechanism of Action	Advantages	Current Status	Disadvantages	References
Fenofibrate	1993	PPAR-α agonist	increased lipoprotein lipase expression	in clinical use	side effects on skeletal muscle, elevation of serum amino- transferase	[7,11,18,33,39]
Volanesorsen	2019 (EU 1/19/1360/001) authorized for the treatment of FCS, declined in US	antisense oligonucleotide, designed to inhibit apo C-III formation	lowering of TGs by over 70%, decreased pancreatitis	in clinical use	injection site reactions, careful monitoring of thrombo-cytopenia and hepatic and renal function	[44]
Evinacumab	2021 (FDA, EU)	human monoclonal antibody that neutralizes angiopoietin-like 3, an inhibitor of lipoprotein lipase	reduced plasma TG and LDL-C in an LDL-receptor-independent manner, used in homozygous familial hypercholesterolemia in which statins are ineffective	in clinical use	possible flu-like illness, nausea, severe allergic reactions	[45]
Alipogene Tipavorvec	2015 (FDA)	adeno-associated virus gene therapy	lipoprotein lipase synthesis and TG reduction	not clinically available	multiple intramuscular injections, lack of long-term efficacy	[34,47,48]
Omega-3s	2000 (EMA)	fatty acid oxidation, decrease in hepatic lipogenesis, stimulation of lipoprotein lipase activity	reduced cholesterol andTGs, anti-inflammatory and antioxidant properties, beneficial effects on endothelial function	no longer considered effective in preventing heart disease	mild side effects: diarrhoea, nausea, dyspepsia, abdominal discomfort	[49,50,53,54,55]
Artichoke leaf extract; Berberine; Bergamot	-	Plasma TG reduction	contributed to the regulation of lipid levels in the blood	in use	few side effects, mainly gastrointestinal	[58,59,61,62,63,64]

## 4. Future Perspectives on Hypertriglyceridemia Therapy

In this section, we describe the potential therapeutic candidates for hypertriglyceridemia therapy (Table 3). Clinical research is ongoing to find new pharmacological options that target novel pathways and overcome the limitations of traditional therapeutic approaches. The described drugs are currently under clinical investigation to verify their efficacy and safety. The results of the studies will be considered to assess if these drugs will be used in therapy and if they will become the future of hypertriglyceridemia therapy. The European Medicines Agency and the Food and Drug Administration in the US are in charge of the approval, registration and monitoring of these pharmaceutical products.

### 4.1. Selective PPARα Activator 

Currently, there is growing interest in the selective activation of PPARα, since older fibrates have relatively weak activity and display limited efficacy. Among fibric acid derivatives, a new selective PPAR-α activator, pemafibrate, has been reported to be markedly effective in reducing triglycerides [57,66]. PPARα activation by pemafibrate was shown to be >2500 times stronger than by fenofibric acid. Pemafibrate produced TG reduction equivalent to, or stronger than, fenofibric acid in rats without leading to changes in liver weight [67]. Analysis of the transcriptome of pemafibrate-regulated genes in primary human hepatocytes and mouse liver indicated that the groups of induced and suppressed genes differed between pemafibrate and fenofibrate [68]. Pemafibrate has a Y-shaped structure, just like the ligand binding domain in PPARα; this results in increased binding affinity of pemafibrate to the entire cavity area. In preclinical studies, pemafibrate was shown to significantly reduce TG levels and increase HDL-C levels compared to fenofibrate. In Sprague-Dawley rats, pemafibrate inhibited VLDL secretion and increased TG clearance through lipoprotein lipase activation. Pemafibrate has been shown to increase VLDL receptor expression, resulting in increased VLDL catabolism. Pemafibrate (1 mg/kg) increased HDL-C levels more than fenofibrate (100 mg/kg) in apo E2 transgenic mice and improved cholesterol efflux from macrophages. It showed a strong anti-inflammatory effect and attenuated atherosclerosis after mechanical damage [69]. Pemafibrate inhibited mRNA expression of the intestinal cholesterol transporter NPC1L1 in the mucosa of the small intestine of mice fed a high-fat diet, eliminating postprandial hyperlipidaemia [70].

Pemafibrate was approved in Japan for the treatment of hyperlipidaemia in June 2017. It has been found to be a substrate of several cytochromes, including CYP2C8, CYP2C9 and CYP3A4, suggesting possible drug interaction [3]. Further studies are planned to gain insights into the possible benefits of this drug. The PROMINENT (Pemafibrate to Reduce Cardiovascular Outcomes by Reducing Triglycerides in Patients with Diabetes) study was specifically designed to test the hypothesis that pemafibrate lowers triglycerides and apo C-III. This drug is currently under investigation in subjects with elevated baseline levels of triglycerides, including diabetic patients with or without recognized coronary artery disease. The primary endpoint is the time of occurrence of the first non-fatal myocardial infarction, ischemic stroke, unstable angina or coronary revascularization and death due to cardiovascular diseases. A significant reduction in triglycerides (26%) occurred after 4 months of treatment compared with baseline, remnant cholesterol (25%) and apo C-III (27%). However, these positive results were counterbalanced by increases in LDL cholesterol and apolipoprotein B and the lack of reduction in HDL cholesterol. Side effects of Pemafibrate included renal adverse events and risk of venous thromboembolism [71]. The PROMINENT trial was designed to consider its future place in therapy. However, the results of the study have greatly reduced the enthusiasm for adding fibrates to statin therapy for cardiovascular events.

### 4.2. Antisense Oligonucleotides Proposed in Therapy

Agents that reduce apo C-III and inhibit ANGPTL3 are currently undergoing clinical evaluation. Vupanorsen is a new therapeutic option, an antisense oligonucleotide that inhibits ANGPTL3 production [72]. In a double-blind phase 2 study (NCT04516291), patients (N = 105) with fasting triglycerides >150 mg/dL, type 2 diabetes and hepatic steatosis were treated for 6 months with 40 or 80 mg every 4 weeks or 20 mg every week with vupanorsen or placebo administered subcutaneously. Efficacy assessment was the percentage change in fasting triglycerides compared to baseline at 6 months. The median TG at baseline was 252 mg/dL. Significant reductions in triglycerides of 36%, 53% and 47% and in ANGPTL3 of 41%, 59% and 56%, respectively, were observed in the 40 mg Q4W (every 4 weeks), 80 mg Q4W and 20 mg QW (once a week) groups compared to the 16% reduction in triglycerides and 8% increase in ANGPTL3 in the placebo. Compared to placebo, vupanorsen 80 mg Q4W reduced apo C-III (58%), residual cholesterol (38%), total cholesterol (19%), non-high-density lipoprotein cholesterol (HDL-C; 18%), HDL-C (24%) and apolipoprotein B (9%) [73]. Despite the decrease in plasma TG, hepatic TG content increased as a compensatory mechanism; serum transaminases also increased; thus, the pharmaceutical company blocked drug development [73]. 

Olezarsen is another antisense oligonucleotide targeting messenger RNA for apo C-III, a genetically validated target for lowering triglyceride [74]. In patients with moderate hypertriglyceridemia and elevated cardiovascular risk and in those with severe hypertriglyceridemia, monthly injections of olezarsen led to significant reductions in triglyceride levels at 6 months compared with placebo (NCT05355402). Olezarsen is similar to volanesorsen but does not adversely affect platelets. A total of 154 patients at 24 sites in North America were recruited. The mean age of the patients was 62 years, and the median triglyceride level was 241.5 mg/dl. The 50 mg and 80 mg doses of olezarsen reduced triglyceride levels by 49.3% and 53.1%, respectively, compared to placebo (*p* < 0.001). Each dose of olezarsen significantly reduced apo C-III, apolipoprotein B and non-HDL cholesterol levels compared to placebo. The risks of adverse events and serious adverse events were similar in the three groups and included liver, kidney or platelet abnormalities [74]. Olezarsen is a GalNAc-conjugated ASO that specifically binds hepatic apo C-III mRNA and reduces apo C-III production and TG levels. As a result, lower doses of olezarsen may be used to reach a clinically substantial effect with the advantage of reduced exposure to extra-hepatic tissues [75,76].

### 4.3. RNA Interference (RNAi)

In a recent randomized controlled trial involving individuals with mixed hyperlipidaemia, plozasiran, as compared with placebo, significantly reduced triglyceride levels. It is based on RNA interference therapy, which is able to silence apo C-III gene expression. Triglyceride-rich lipoproteins are downregulated due to apo C-III-mediated inhibition of lipoprotein lipase. A clinical trial (NCT04998201) is ongoing [77]. This study involves persons with mixed hyperlipidaemia at risk of atherosclerotic cardiovascular disease due to elevated cholesterol levels and triglyceride-rich lipoproteins [78]. zodasiran is also an RNA interference (RNAi) therapy targeting *ANGPTL3* mRNA expression in the liver. In patients with mixed hyperlipidaemia, zodasiran was associated with significant decreases in triglyceride levels (NCT04832971) [79]. It is useful in ANGPTL3 loss-of-function carriers that have lower levels of triglycerides and low-density lipoprotein (LDL) cholesterol who are at risk of atherosclerotic cardiovascular disease with respect to the non-carriers [72]. 

### 4.4. Analog of Human Fibroblast Growth Factor 21

A new therapeutic proposal includes pegozafermin, a glycopegylated recombinant analogue of human fibroblast growth factor 21 (FGF21) designed to have a longer half-life with respect to the native FGF21, showing the same effect of the native hormone on its receptor [80]. FGF21 is an endogenous stress hormone that regulates lipid and glucose metabolism as well as energy expenditure and pegozafermin is being developed for the treatment of severe hypertriglyceridemia and non-alcoholic steatohepatitis (NASH). This can be an ideal therapy in light of the benefit it can produce for other metabolic-associated conditions such as obesity, metabolic syndrome, insulin resistance, type 2 diabetes mellitus (T2DM) and non-alcoholic fatty liver disease (NAFLD). Indeed, pegozafermin demonstrated beneficial effects on serum lipids such as TGs, LDL cholesterol and HDL cholesterol, as well as insulin resistance, body weight and liver fat in patients with NASH [81]. Pegozafermin and other FGF21 analogues have already demonstrated improvements in lipid management in both healthy volunteers and patients with diabetes or NASH; now, the effects of this treatment are under investigation as a new therapeutic compound for the treatment of severe hypertriglyceridemia [54]. Preclinical data suggest that FGF21 stimulation reduces fat in the liver via increased AMPK signalling, which stimulates fatty acid oxidation, and triglyceride elimination. Remarkably, FGF21 increases low-density lipoprotein receptor (LDLR) expression, which could raise LDL and VLDL uptake (NCT04929483) [82,83].

**Table 3:** Potential therapeutic candidates for hypertriglyceridemia therapy.

Drug	Mechanism of Action	Advantages	Current Status	Disadvantages	References
Pemafibrate	selective PPAR-α agonist	high selectivity of the receptor, more effective than fenofibrate	under investigation	renal adverse events and risk of venous thromboembolism	[3,57,66]
Vupanorsen	antisense oligonucleotide inhibits ANGPTL3 production	reduction in cholesterol and plasma TGs	discontinuation of the clinical development programme	increase in serum transaminases	[57,72,73]
Olezarsen	antisense oligonucleotide targeting messenger RNA of apo C-III, responsible for the inhibition of lipoprotein lipase	significant reductions in TGs levels, does not adversely affect platelets	Phase III NCT05355402	liver and kidney abnormalities	[74,75,76]
Plozasiran	siRNA silencing of apo C-III gene expression, which is responsible for the inhibition of lipoprotein lipase	decrease in TGs levels	a clinical trial is ongoing (NCT04998201)	favourable safety profile	[77,78]
Zodasiran	siRNA targeting ANGPTL3	decrease in TGs levels	under investigation	transient elevation in glycated haemoglobin levels in patients with pre-existing diabetes who received the highest dose	[72,79]
Pegozafermin	analogue of FGF21	reduction in TGs and LDL cholesterol	under investigation	long-term data on safety are needed	[80,81,82,83,84]

## 5. Conclusions

**Figure 2:**
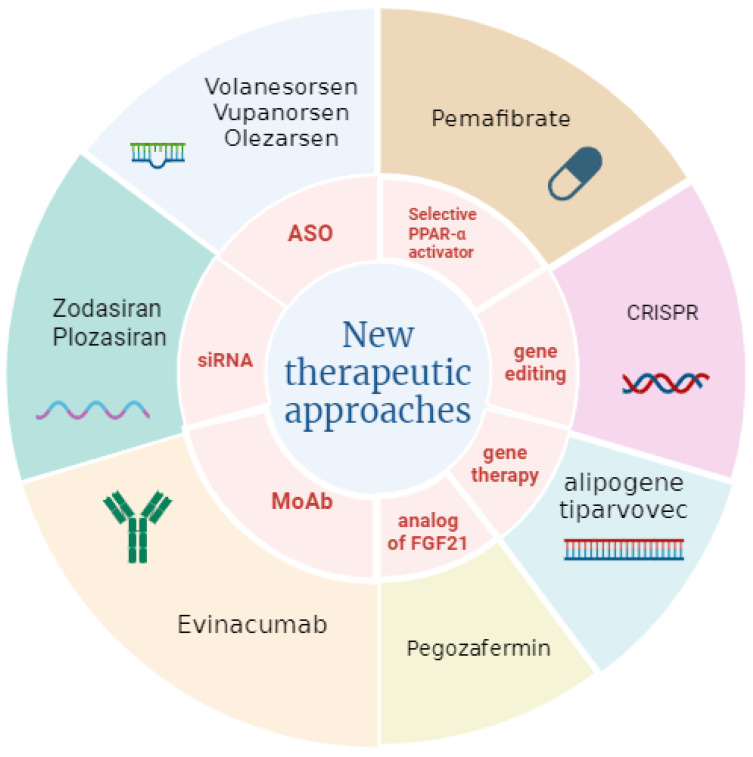
Description of recent therapeutic approaches that are useful for reducing hypertriglyceridemia, a disease that can be responsible for the progression of cardiovascular diseases. The drugs (already used in therapy or in clinical trial evaluations) are classified based on their mechanisms of action. See text for details. ASO: antisense oligonucleotides; MoAb: monoclonal antibody; FGF21: human fibroblast growth factor 21.

We are now entering a new era of lipid-lowering drugs. Figure 2 summarizes the present and future therapies for hypertriglyceridemia. Efforts to personalize therapy and target the right patient at the right time include further refinement of risk stratification tools, genetic aspects consideration and pharmacological approaches to define drug efficacy as well as primary and secondary clinical outcomes [84]. A new approach for the treatment of hypercholesterolaemia is the CRISPR-Cas9 system, which allows specific DNA sequences to be cut by modifying or deactivating target genes. Recent studies have shown that it is possible to use CRISPR-Cas9 to deactivate genes such as proprotein convertase subtilisin/kexin type 9 (*PCSK9*), which regulate LDL cholesterol levels. Deactivating *PCSK9* leads to a significant reduction in bad cholesterol in the blood. Proof-of-concept studies of in vivo somatic genome editing in mice have highlighted the therapeutic potential of CRISPR-Cas9 to target PCSK9 [85]. Future studies will allow the application of this technology to hypertriglyceridemia. 

## Figures and Tables

**Table 1:** Past therapies for hypertriglyceridemia.

Drug	Year of Approval	Mechanism of Action	Advantages	Current Status	Disadvantages	References
Clofibrate	1967	PPAR-α agonist	reduced risk of heart disease	not in use	side effects on skeletal muscle, liver, gastrointestinal tissue, gallstones	[5,7]
Gemfibrozil	1981	PPAR-α agonist	reduced risk of heart disease	in clinical use	increased risk of pancreatitis	[9]
Niacin	1955	inhibition of triglyceride synthesis	reduced atherogenic lipoprotein particles	not in use in the EU	serious liver toxicity	[33,34]
